# Epstein-Barr virus-coded miR-BART19-3p promotes proliferation of EBV-associated gastric cancer by inhibiting GADD45B

**DOI:** 10.1186/s12967-025-06955-9

**Published:** 2025-08-25

**Authors:** Jing Yang, YueRu Han, Bin Zeng, Guangsheng Hu, Zhifeng Liu, Runliang Gan

**Affiliations:** 1 Department of Gastroenterology, The First Affiliated Hospital of University of South China, Hengyang, 421001 Hunan Province China; 2 Cancer Research Institute, Hunan Province Key Laboratory of Tumor Cellular and Molecular Pathology, University of South China, Hunan, 421001 China; 3 Department of Otorhinolaryngology, The First Affiliated Hospital of University of South China, Hengyang, 421001 Hunan Province China

**Keywords:** EBV-associated gastric cancer, Epstein-Barr virus-coded miR-BART19-3p, GADD45B, Proliferation

## Abstract

**Background:**

EBV-associated gastric cancer (EBVaGC) is a distinct molecular subtype of gastric cancer with EBV latency infection. EBV microRNAs, especially rightward transcript (BART) microRNAs, play key roles in the tumor growth of EBVaGC. However, the effects of EBV-miR-BART19-3p in EBVaGC remain largely unknown.

**Methods:**

EBV-miR-BART19-3p was detected by qRT-PCR in EBER in situ hybridization positive gastric carcinoma tissues. CCK8, Colony formation, EdU and Flow cytometry were conducted in gastric cancer cells with overexpression and knockdown of EBV-miR-BART19-3p compared to the negative control, respectively. Tumor xenografts were conducted in vivo. GADD45B was screened by RNA sequencing and dual-luciferase reporter assays implemented with transfecting EBV-miR-BART19-3p antagomir. Proteins were confirmed by Western blotting.

**Results:**

EBV-miR-BART19-3p was highly expressed in EBVaGC and promoted the proliferation of EBV-associated gastric cancer cells in vitro and accelerated xenograft tumor growth in vivo. Knockdown of EBV-miR-BART19-3p induced cell cycle G2/M phase arrest in AGS EBV cells. Molecularly, EBV-miR-BART19-3p directly targets the 3′-UTR of growth arrest and DNA damage-inducing protein Beta (GADD45B) mRNA and downregulates its protein expression, which consequently reduced cell cycle G2/M phase arrest. Reconstitution of GADD45B can rescue the proliferation phenotype caused by EBV-miR-BART19-3p in EBVaGC cells.

**Conclusions:**

Our study indicated Epstein-Barr Virus MicroRNA BART19-3p played an oncogenic role by a novel mechanism of inhibiting GADD45B in EBVaGC, which provides new insights and might be a potential therapeutic strategy for EBVaGC patients.

**Supplementary Information:**

The online version contains supplementary material available at 10.1186/s12967-025-06955-9.

## Introduction

Gastric cancer (GC) is a common malignancy, which ranks fifth for the annual incidence and fourth for the cancer mortality according to the 2020 global cancer statistics [1]. The Cancer Genome Atlas (TCGA) had performed detailed bioinformatics exploration on the genetic sequencing data of gastric cancer patients and introduced a novel molecular classification of gastric cancer: chromosomal instability, Epstein-Barr virus (EBV) positive, genomically stable, and microsatellite unstable tumors [2]. EBV positive gastric cancer is also called EBV-associated gastric cancer (EBVaGC). EBVaGC accounts for approximately 1.3–30.9% of all gastric cancers with variations across geographical regions [3–5], with approximately 75,000 new cases every year worldwide [6]. However, the mechanism of tumorigenesis and progression of EBVaGC is not clear. Hence, it is of great clinical significance to mine new molecular biomarkers and therapeutic targets for EBVaGC.

EBV is an oncogenic virus associated with multiple malignant tumors, including NK/T-cell lymphoma, Hodgkin, Burkitt, leiomyosarcoma, nasopharyngeal carcinoma, and gastric cancer [7–9]. EBV was the first virus discovered to express miRNA [10]. EBV Bam-HI-A rightward transcripts (EBV BART miRNAs) are key molecules during the pathogenesis of EBV-related tumors, including immune evasion, cell proliferation, apoptosis and tumor metastasis [11–15]. EBV BART miRNAs encode 25 pre-miRNA precursors and 44 mature miRNAs. EBV BART miRNAs can be detected in all types of EBV-associated latency tissues [16, 17] and were highly expressed in infected epithelial tissues [18]. Interestingly, EBVaGC belonging to type I or type II latency rarely expresses the EBV classic oncoprotein LMP1 but highly expresses a large amount of EBV BART microRNA [19–21], which attracts our attention.

MicroRNAs (miRNAs) are short non-coding RNAs constituted of approximately 22 nucleotides, which bind to the 3’UTR of target mRNAs and result in inhibiting their protein translation or destabilizing the target mRNAs according to the degree of sequence complementarity between miRNA and its target [22–24]. Recent studies have shown that BART miRNAs can target the mRNA of epithelial host cells to promote host cell survival and tumor metastasis by EMT in nasopharyngeal carcinoma [25–28]. However, their roles in oncogenesis and growth of EBVaGC remain unclear; more in-depth research is needed to elucidate their role in gastric cancer.

This study reveals that EBV-miR-BART19-3p is highly expressed in EBVaGC tissues and promotes the proliferation of EBVaGC in vivo and in vitro. Through sequencing analysis, we further found that EBV-miR-BART19-3p promoted tumors in EBVaGC by directly targeting GADD45B to reduce cell cycle G2/M phase arrest, which may afford novel targets for the treatment of EBVaGC. Our findings exhibit new insights into the role of EBV-miR-BARTs in the pathogenesis of EBVaGC.

## Materials and methods

### Cell lines and culture

GES1 (Immortalized EBV-negative gastric epithelial cell line), SGC-7901 (EBV-negative gastric cancer cell line), and Raji (EBV positive Burkitt lymphoma cell line) were obtained from University of South China and cultured with RPMI 1640 (Gibco). AGS (EBV-negative gastric cancer cell line, ATCC CRL-1739) was cultured with Ham’s F-12 medium (Hyclone). HEK293T was cultured with DMEM (Gibco). All above culture mediums were supplemented with 10% fetal bovine serum (FBS, Gibco), 1% penicillin/streptomycin (Hyclone). AGS-EBV (EBV-positive gastric cancer cell line) was cultured in Ham’s F-12 medium with 10% fetal bovine serum and additionally 400 μg/ml of G418 to retain the EBV genome.

### Patient specimens

95 surgical gastric cancer specimens and paired normal gastric mucosal tissue were collected from the First Affiliated Hospital of University of South China (Hengyang, China). Specimens were preserved in liquid nitrogen and formalin-fixed for the subsequent experiments. Informed consent was obtained from all patients with approval by the medical ethical review committees of the First Affiliated Hospital of University of South China. Clinical and pathological data were collected. All GC patients were diagnosed by pathological examinations, and EBVaGCs were regarded as positive by EBER in situ hybridization (ISH).

### EBER-ISH and immunohistochemical staining

EBER-ISH was conducted in the paraffin-embedded tissue sections using the EBER1 probe from the ISH kit (SH-7001, Zsbio, China). The steps were followed according to kit protocol. Samples with universal brown nuclear staining in cells were elucidated as EBER positivity.

Paraffin-embedded sections from nude mice were applied to hematoxylin and eosin (H&E) staining and immunohistochemical (IHC) staining for the detection of protein levels of proliferation markers. The streptavidin-peroxidase method was used under kit protocol (PV-9000, Zsbio, China). Ki67 and PCNA were both localized in the nucleus.

### PCR and real-time PCR

DNA extraction was performed utilizing TIANamp Genomic DNA Kit (DP304, TIANGEN, China). 1μg isolated template DNA from patient tissue or cells for detecting interesting genes was amplified by PCR using Premix TaqTM Mix (RR902A, Takara, Dalian) following the manufacturer’s protocol. PCR products were visualized by 2% agarose gel in TAE buffer.

Total RNAs were extracted by TRIzol reagent (Invitrogen). According to the miRNA 1st Strand cDNA synthesis kit (Thermo Scientific, USA), 1 μg of RNA was required to create cDNA for miRNA reverse transcription. For miRNA reverse transcription, cDNA was synthesized with 1μg RNA and a miRNA 1st Strand cDNA synthesis kit (MR101, Vazyme, China) utilizing stem-loop assay. Quantitative real-time PCR (qPCR) was executed using Maxima SYBR Green/ROX qPCR Kit (Thermo Scientific, USA) and the miRNA universal SYBR qPCR master mix kit (MQ101, Vazyme, China) according to the manufacturer's protocol, respectively. The expression levels of miRNA and mRNA were normalized by U6 and GAPDH, respectively. Quantification of gene transcripts was conducted with triplicate SYBR microRNA assays by the 2-△△CT method. The primers of genes are listed in Supplementary Table S1.

### Transient transfection and Lentivirus transfection

The EBV-miR-BART19-3p mimics and antagomir were obtained from Ribobio Inc. (Guangzhou, China). Sequences were listed in Supplementary Table S2. The transfection was performed with 50 nM mimics or 100 nM antagomir utilizing Lipofectamine 3000 (Invitrogen) for 48 h before confirmed by qPCR.

Lentiviral particles (GV309, hU6-MCS-Ubiquitin-EGFP-IRES-puromycin) containing EBV-miR-BART19-3p precursor (BART19-3p for short) and its randomized flanking sequence control (NC for short) were purchased from GeneChem (Shanghai, China). AGS and GES-1 cells were transfected using lentiviral particles according to the manufacturer’s protocol (MOI = 10). Subsequently, the cells were filtered with 2μg/uL of Puromycin for 7 days after 72 h of transfection. The EBV-miR-BART19-3p stable-expression cells were validated by qPCR and were identified as AGS-LV-BART19-3p and GES1-LV-BART19-3p, respectively.

### CCK8, colony formation, and EdU

For CCK8 Cell proliferation analyses, 3000 cells were cultured in 96-well plates and cell viability was detected at 0 h, 24 h, 48 h, 72 h and 96 h after different treatments by CCK8 solution (A311-02, Vazyme, China). For colony formation, 800 cells were seeded into 6-well plates for 12 days and subsequently washed with phosphate-buffered saline (PBS), fixed with 0.4% formaldehyde, and stained with crystal violet. Colonies containing more than 50 cells were recorded. EdU was performed 12 hours after cell seeding using BeyoClick™ EdU-594 (Beyotime, China) with a 2-hour incubation period, following the manufacturer’s instructions.

### Flow cytometry

Cells were transfected with miRNA antagomir or NC in 6-well plates for 48 h. For cell cycle, cells were collected and fixed in ice-cold 75% ethanol for 6 h at 4 °C. Then cells were washed twice by PBS, incubated using 100 μg/ml RNase A (Takara, Dalian) for 10 min at 37 °C, and stained by 50 μg/ml propidium iodide (BD PharmingenTM, USA) in dark at 25 °C for 30 min. Stained cells were analyzed utilizing flow cytometry. For cell apoptosis detection, the number of apoptotic cells was detected using Annexin V-FITC/PI apoptosis detection kit (556547, BD PharmingenTM, USA) following the manufacturer’s instructions.

### Plasmids and dual-luciferase reporter assays

3′UTR luciferase reporter assays were conducted using the pmirGLO vector, the dual-luciferase assay kit from Promega (Madison, USA) that expresses both firefly luciferase and Renilla luciferase. HEK293T cells were co-transfected utilizing 50 nM EBV-miR-BART19-3p mimic or NC, and 200 ng of pmirGLO vector that contained the wild-type or mutant 3’UTR of GADD45B. Afterward, the luciferase activities were measured using a fluorescence detector (Modulus, USA) and analyzed using a dual-luciferase reporter assay. Transfections were repeated in three independent experiments.

### Tumor xenografts in nude mice

All BALB/c nude mice (4–5 weeks old, female) were procured from Hunan SJA Laboratory Animal Co. Ltd (Changsha, China). All animal study plans were approved by the Animal Ethics Committee of University of South China. To assess the role of EBV-miR-BART19-3p in tumor growth in vivo, 200 μL of AGS-LV-BART19-3p cells or control cells (4 × 10^6 cells per mouse) were subcutaneously inoculated into the left or right back of each mouse. The volume of tumor was calculated by the formula: (L × W^2^)/2, in which L and W represent the long and short diameters of the tumor, respectively. 12 days after tumor formation, the mice were randomly separated into 2 groups (each five mice): one group was subjected to local injection of 10 nmol EBV-miR-BART19-3p antagomir into the tumor mass every 3 days for 4 times; the other group was locally treated using equal volumes of physiological saline. All nude mice were euthanized 22 days after cancer cell inoculation.

### RNA sequencing and data analysis

Total RNAs of AGS EBV cells transfected with EBV-miR-BART19-3p antagomir and NC antagomir were isolated using TRIzol (Invitrogen). Then, the quality of RNA was checked using an Agilent2100 RNA Nano 6000 Assay Kit (Agilent Technologies, CA, USA). RNA sequencing was conducted on the DNBSEQ platform by BGI Genomics (Shenzhen, China). Subsequent data analysis, the differentially expressed genes (DEGs), Gene Ontology (GO), and Kyoto Encyclopedia of Genes and Genomes (KEGG) were performed with our previous studies [29, 30].

### Western blotting

Protein extracts were separated in 8–15% SDS–polyacrylamide gel and blotted onto polyvinylidene difluoride (PVDF) membranes. PVDF Membranes were probed utilizing antibodies against GADD45B (1:500; Abcam), PCNA (1:2000; Proteintech), Ki67 (1:1000; Proteintech), cyclin B1 (1:2000; Proteintech), CKD1 (1:2000; Proteintech), CCDC80 (1:1000; Immunoway), FosB (1:2000; Immunoway), Tubulin (1:5000; Abbkine) and then incubated with horseradish peroxidase modified secondary antibody (Thermo Scientific, USA). Protein bands were visualized using enhanced chemiluminescence (ECL) substrate (Pierce, Thermo Scientific). Western blot analysis and quantification were performed using ImageJ software.

### Statistical analysis

Experimental data were described by SPSS 22.0 and analyzed by GraphPad Prism 7. Data are presented as mean ± standard deviation (SD) of the three independent experiments or presented as quartiles if nonnormal distribution. The t-test and one-way ANOVA were used to determine the differences between two groups and three or more groups, respectively. If the data did not obey normal distribution, a non-parametric rank-sum test was used. Fisher's exact test was used for the clinical data of patients. *p* < 0.05 (labeled as *), *p* < 0.01 (labeled as **) and *p* < 0.001 (labeled as ***) were considered a statistically significant.

## Results

### EBV-miR-BART19-3p is highly expressed in EBVaGC tissues and AGS EBV cell lines

**Fig. 1:**
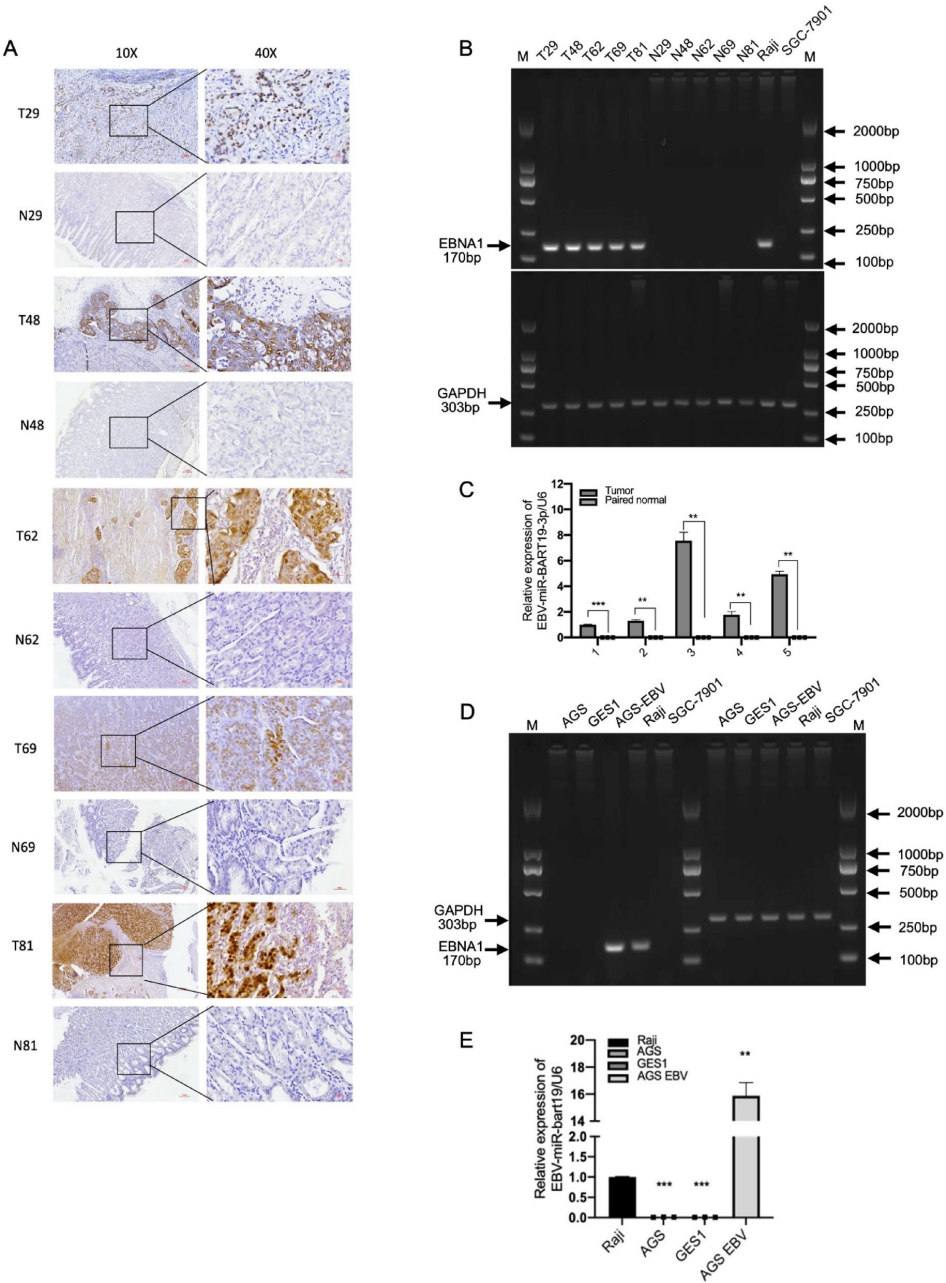
EBV-miR-BART19-3p is highly expressed in EBVaGC tissues and AGS EBV cell lines. (**A**) EBER-ISH was used to detect EBV infection in 95 gastric cancer tissues, and typical pictures of EBER-ISH results of cancer tissues and paired normal gastric mucosa of 5 EBV-positive gastric cancer patients were shown, Original magnification: × 100 (left), × 400 (right); scale bar: 100um(left panel), 30um(right panel). (**B**) DNA was extracted from gastric cancer tissues of patients with EBV-related gastric cancer and paired normal gastric mucosa tissues, and EBNA1 (upper, 170 bp) was detected by PCR gel electrophoresis, and GAPDH was used as an internal reference (lower, 303 bp). (**C**) RT-PCR detection of EBV-miR-BART19-3p expression levels in fresh frozen gastric cancer tissues and matched normal gastric mucosa tissues from 5 EBVaGCs. (**D**) DNA of each cell line was extracted, and EBNA1 (170 bp) in AGS, GES1, SGC-7901, AGS-EBV, and Raji cell lines was detected by PCR gel electrophoresis, and GAPDH (303 bp) was used as an internal reference. (**E**) RNA was extracted from each cell line, and RT-qPCR was used to detect the expression of AGS, GES1, AGS-EBV, and EBV-miR-BART19-3p in Raji cells. Paired t-test and one-way ANOVA test analysis, data are shown as mean ± SD of three experiments, **p < 0.01, ***p < 0.001

In this study, we initially collected tissue samples from 95 gastric cancer (GC) patients. 5 cases of EBVaGC were identified by EBER in situ hybridization assay (Fig. 1A). To further confirm EB virus infection state in gastric cancers and their paired normal gastric mucosa tissues in 5 cases of EBVaGC, EBNA1(170 bp) was detected positive in gastric cancer tissues however negative in paired normal gastric mucosa tissues by PCR gel electrophoresis (Fig. 1B). Then, we detected EBV-miR-BART19-3p in the 5 cases of EBVaGC. EBV-miR-BART19-3p was upregulated in EBVaGC specimens compared with the paired normal gastric mucosa tissue specimens by stem-loop qPCR (Fig. 1C). To investigate the relationship of the EBV-miR-BART19-3p with pathoclinical parameters of GC, clinicopathological profile of 95 gastric cancer patients is shown in Supplementary Table S3. The results suggest there were no significant differences with age, gender, tumor size, histologic differentiation, and TMN stages in EBVaGC and non-EBVaGC. Using EBV-positive lymphoma cells Raji as a positive control and gastric cancer SGC-7901 cells as a negative control, PCR gel electrophoresis showed that EBNA1, a marker of EBV infection, was negative in AGS, SGC-7901, and GES1 cell lines, but positive in AGS-EBV and Raji cell lines (Fig. 1D). The level of EBV-miR-BART19-3p in the AGS EBV cell lines was significantly higher than AGS and GES1 cell lines with Raji used as a standard by RT-qPCR (Fig. 1E).

### Overexpression of EBV-miR-BART19-3p endorses proliferation of AGS and GES1 cell lines in vitro

**Fig. 2:**
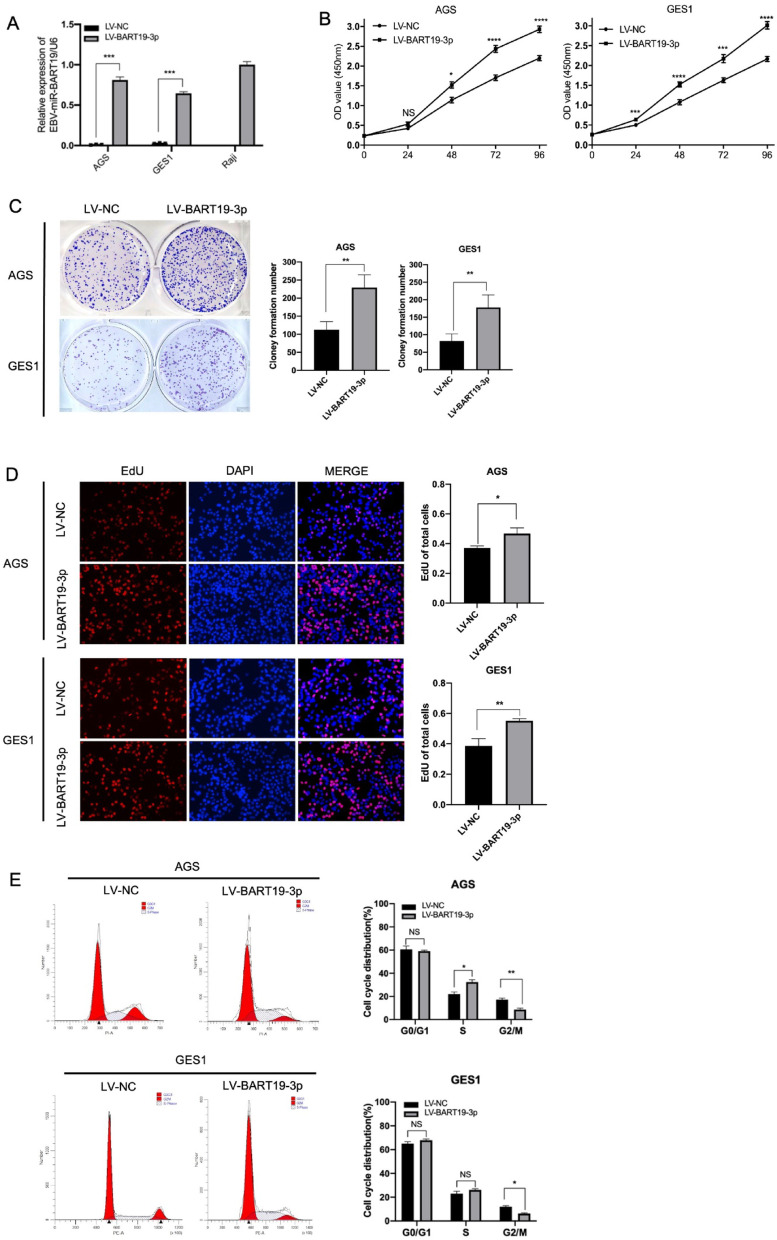
Overexpression of EBV-miR-BART19-3p promotes proliferation of AGS and GES1 cell lines in vitro. (**A**) Stable BART19-3p over-expressing cell lines were constructed by lentiviral transfection in AGS and GES1. The expression of EBV-miR-BART19-3p was detected by RT-qPCR with EBV-positive lymphoma Raji cells as a positive reference. (**B**) The proliferation ability of AGS LV-BART19-3p stably transfected cell line and GES1 LV-BART19-3p stably transfected cells were detected by CCK8 assay compared to the negative control. (**C**) Colony-forming assays were performed after AGS and GES1 cell lines were transfected by the NC or BART19-3p lentiviral vector respectively. (**D**) DNA replication ability was detected by EdU incorporation assay. (**E**) Cell cycle distributions of LV-BART19-3p and LV-NC in AGS stable cell lines were detected by flow cytometry after PI staining

To study the function of EBV-miR-BART19-3p, we established AGS and GES1 cell lines that stably overexpressed EBV-miR-BART19-3p by lentiviral transfection (Supplementary Fig. 1) with the expression level of EBV-miR-BART19-3p in Raji cell line as a reference (Fig. 2A). CCK8 (Fig. 2B) assay and clone formation (Fig. 2C) showed that EBV-miR-BART19-3p elevation endorsed AGS and GES1 cells proliferation Then we examined the function of EBV-miR-BART19-3p on DNA replication, cell cycle distribution and cell apoptosis ratio by analyzing the incorporation of EdU into cellular DNA and flow cytometry analysis. EdU assay revealed that EBV-miR-BART19-3p facilitated DNA replication activity (Fig. 2D). Additionally, compared to the negative control cells, cells overexpressing EBV-miR-BART19-3p displayed a reduced period of the G2/M phase in cycle distribution within both AGS and GES1 cell lines and a longer S phase only in AGS cell lines (Fig. 2E). However, overexpression of EBV-miR-BART19-3p did not affect apoptosis rate of cells (Supplementary Fig. 2).

### Knockdown of EBV-miR-BART19-3p inhibits the proliferation of EBV-positive gastric cancer cells in vitro

**Fig. 3:**
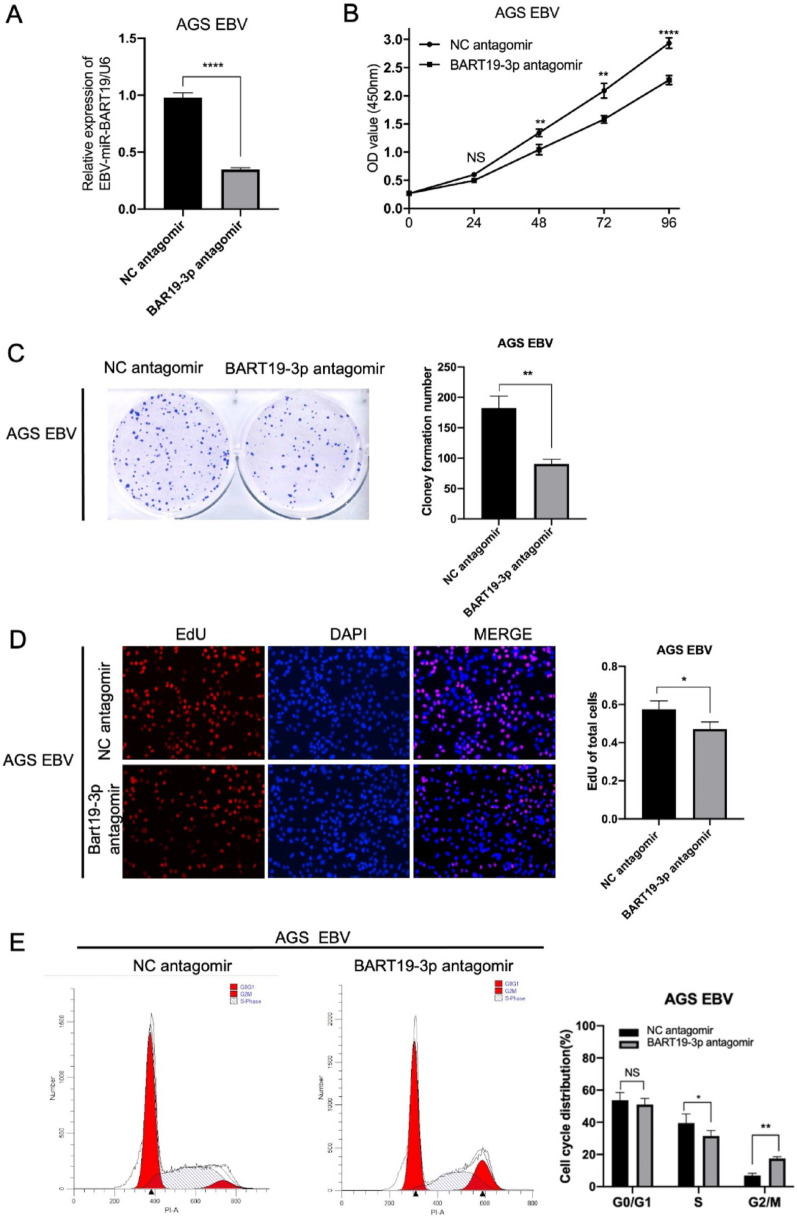
Knockdown of EBV-miR-BART19-3p inhibits the proliferation of EBV-positive gastric cancer cells in vitro. (**A**) The knockdown efficiency of EBV-miR-BART19-3p in AGS-EBV cells was confirmed by RT-qPCR 48 h after transfection with a negative control (NC) or EBV-miR-BART19-3p-specific antagomir (100 nM). The effect of EBV-miR-BART19-3p knockdown on cell proliferation was assessed using the CCK-8 assay (**B**), colony formation assay (**C**), and EdU incorporation assay (**D**) 48 h post transfection with NC or EBV-miR-BART19-3p antagomir (100 nM). (**E**) The cell cycle distribution of transfected AGS-EBV cells was analyzed by flow cytometry following propidium iodide (PI) staining. Three independent experiments were performed. Data are presented as the mean ± SD. *p < 0.05, **p < 0.01, ***p < 0.001, ****p < 0.0001

To further validate the role of EBV-miR-BART19-3p in cell proliferation and cell cycle, we downregulated the level of EBV-miR-BART19-3p in AGS EBV by transfecting antagomir oligonucleotides (chemically modified miRNA antagonists) (Fig. 3A), and the cell growth activity (Fig. 3B) and clone formation ability were decreased (Fig. 3C). EdU incorporation experiments displayed a reduced number of Edu-positive cell after EBV-miR-BART19-3p attenuation (Fig. 3D). Cells transfected by EBV-miR-BART19-3p antagomir exhibited a higher proportion in G2/M phase and a lower proportion in S phase of cell cycle distribution compared with the negative control (Fig. 3E).

### EBV-miR-BART19-3p promotes gastric cancer growth in vivo

**Fig. 4:**
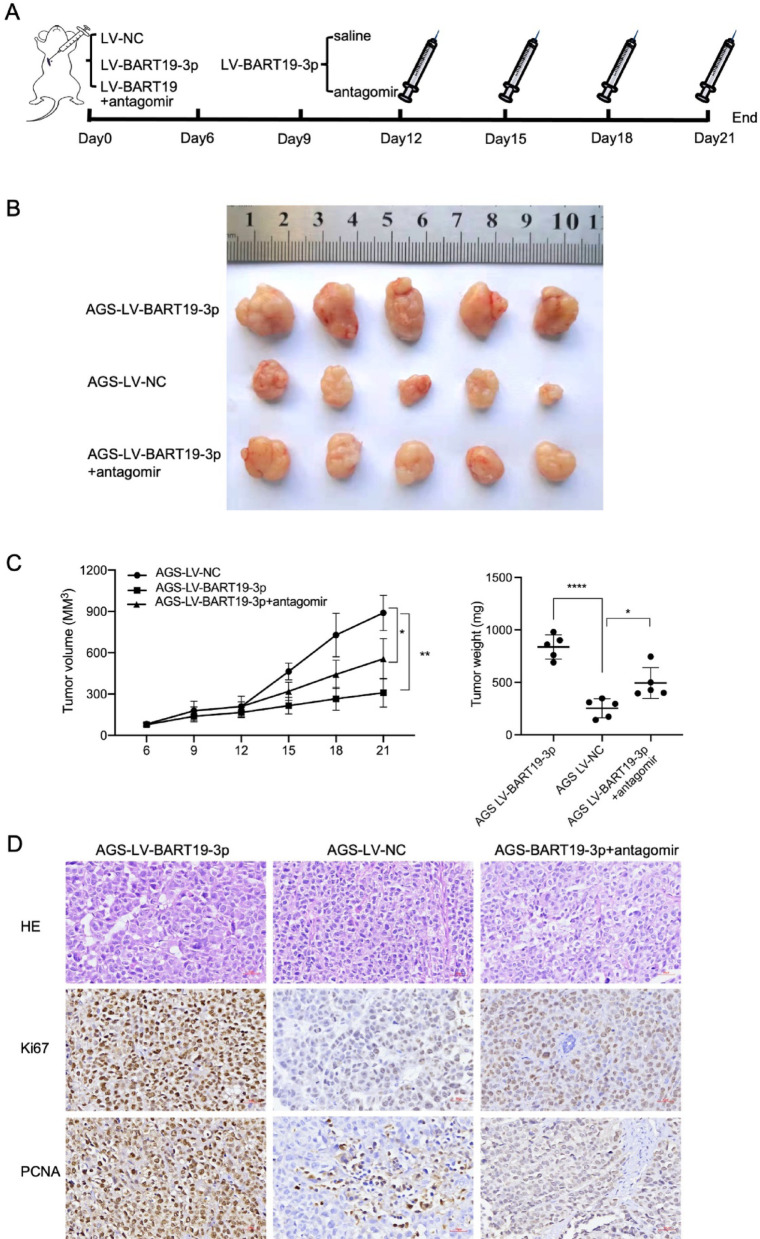
Overexpression of EBV-miR-BART19-3p promotes gastric cancer growth and Knockdown of EBV-miR-BART19-3p inhibits the proliferation of EBV-positive gastric cancer cells in vivo. (**A**) Flow chart of AGS xenografts model in nude mice. Nude mice were subcutaneously injected with LV-NC, and LV-BART19-3p AGS cells respectively then treated with antagomir (see Materials and Methods for details). Xenograft tumors (**B**), tumor growth curves and tumor weight (**C**) were measured on day 21 after subcutaneous implantation (n = 5). (**D**) The representative immunohistochemistry images for Ki67 and PCNA of xenograft tumors were shown. Original magnification: × 100 (left), × 400 (right); scale bar: 100um (left panel), 30um (right panel)

Then, xenograft growth assays were conducted. Figure 4A is a flow chart showing xenograft and intratumoral injection. AGS cells stably overexpressing EBV-miR-BART19-3p (AGS-LV-BART19-3p) and its negative control cells (AGS-LV-NC) were inoculated into nude mice. EBV-miR-BART19-3p overexpression impelled the tumor growth of the AGS-LV-BART19-3p compared to the AGS-LV-NC. Downregulation of AGS-LV-BART19-3p by antagomir attenuated the tumor growth and tumor size (Fig. 4B, C). Furthermore, immunohistochemical (IHC) staining on xenograft tumors exhibited that the elevation of EBV-miR-BART19-3p upregulated the level of Ki67 and PCNA in AGS-LV-BART19-3p tumors. Similarly, downregulation of EBV-miR-BART19-3p by intratumoral injection of antagomir decreased the expression of Ki67 and PCNA (Fig. 4D). Taken together, these data showed that EBV-miR-BART19-3p promotes gastric cancer growth in vivo.

### EBV-miR-BART19-3p directly targets GADD45B mRNA and accelerates Gadd45b protein degradation

**Fig. 5:**
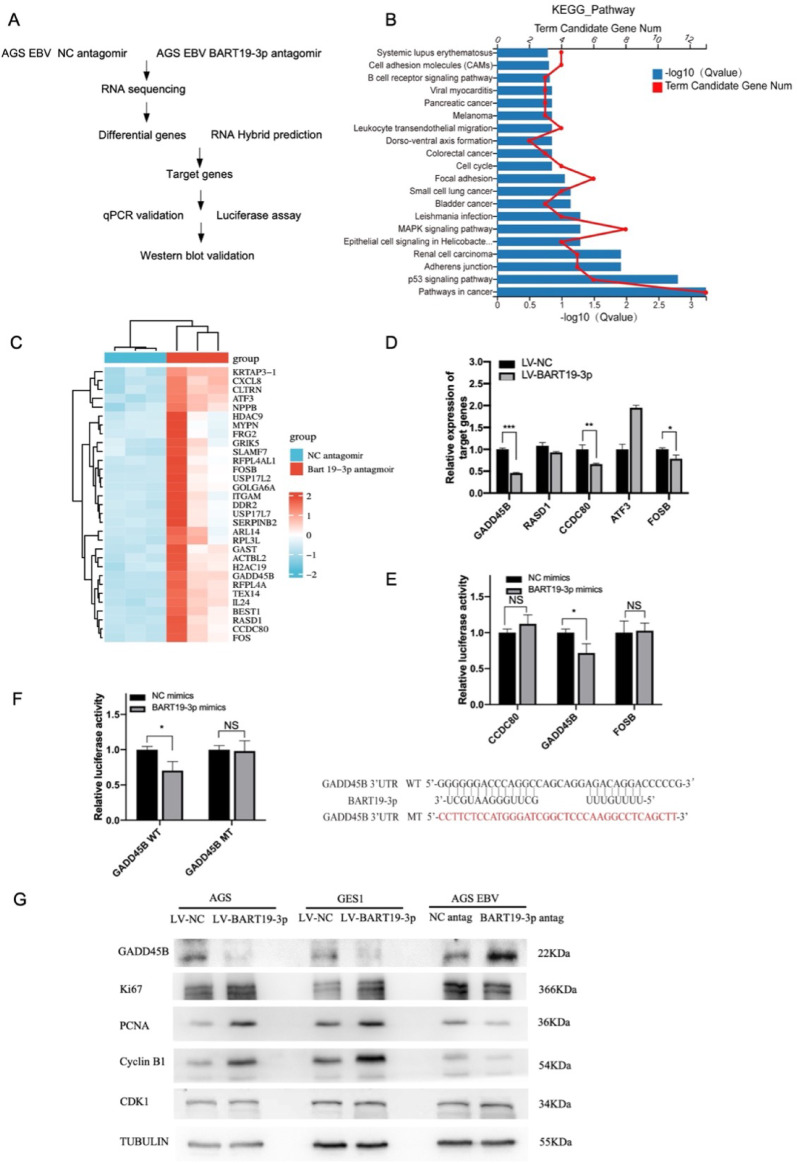
EBV-miR-BART19-3p directly targets GADD45B. (**A**) Flow chart of the integrated analysis for screening targeted genes of EBV-miR-BART19-3p. (**B**) KEGG pathway enrichment analysis of upregulated genes after EBV-miR-BART19-3p knockdown in AGS EBV cells by EBV-miR-BART19-3p antagomir. (**C**) The top 30 protein-coding genes of upregulated genes were displayed by clustering heatmap. Red indicates high expression and blue indicates low expression. Red frame displays five candidate genes predicted from the RNAhybrid website that could interact with EBV-miR-BART19-3p. (**D**) The mRNA levels of GADD45B, FOSB, RASD1, ATF3, and CCDC80 were detected by RT-qPCR after stably overexpressing EBV-miR-BART19-3p in AGS cells. (**E**) Dual-luciferase was performed 36 h after HEK293 cells co-transfected with NC (or EBV-mir-BART19-3p) mimics and CCDC80, GADD45B, FOSB dual-luciferase reporter plasmid, respectively. (**F**) The dual-luciferase was performed 36 h after HEK293 cells co-transfected with NC (or EBV-mir-BART19-3p) mimics and GADD45B dual-luciferase reporter plasmid. (**G**) Protein levels of GADD45B, Ki67, PCNA, cyclin B1, and CDK1 were detected by Western blot with Tubulin as an internal reference. Data are shown as mean ± SD of three experiments, *p < 0.05, **p < 0.01, ***p < 0.001

The above data verified that EBV-miR-BART19-3p endorses gastric cancer cell proliferation in vitro and in vivo. We further investigated how EBV-miR-BART19-3p targeted genes could enhance the proliferation of EBVaGC by an integrated analysis (Fig. 5A). Firstly, Deep RNA sequencing of AGS EBV BART19-3p antagomir cells versus AGS EBV NC antagomir cells identified 206 upregulated genes and 99 downregulated genes, from which significantly difference of 73 up-regulated genes (Table S4) and 1 down-regulated gene were screened with log_2_FoldChange > 2 times and *p* adj < 0.05. KEGG enrichment analysis of upregulated genes showed that EBV-miR-BART19-3p regulated some pathways, including cell cycle, MAPK signaling pathway, pathway in cancer, and P53 signaling pathway (Fig. 5B). The Top 30 upregulated protein coding differential genes are showed in the heatmap (Fig. 5C). Then, we screened target genes of EBV-miR-BART19-3p by RNAhybird (https://bibiserv.cebitec.uni-bielefeld.de/rnahybrid/submission.html/) from these 30 top differential genes. Based on the predictive possibility of miRNA binding to mRNA target and their minimum free energy (MFE), five candidate target genes were focused on: GADD45B, FOSB, RASD1, ATF3, and CCDC80. We further confirmed with RT-qPCR that the mRNA expressions of GADD45B, FOSB, and CCDC80 were reduced after overexpression of EBV-miR-BART19-3p (Fig. 5D).

To clarify whether EBV-miR-BART19-3p can directly bind to the target gene, we performed preliminary dual-luciferase reporter assays. The activity of luciferase containing the wild-type GADD45B 3’-UTR (GADD45B WT) but not FOSB and CCDC80 was significantly reduced by EBV-miR-BART19-3p mimics (Fig. 5E). RNAhybrid analysis suggested that the GADD45B 3′-UTR region was base paired with the seed sequences of EBV-miR-BART19-3p in the nucleotide position 946. Next, a GADD45B 3′-UTR mutant (GADD45B MT) vector was constructed to break the base binding. The luciferase activity of GADD45B WT but not GADD45B MT was significantly inhibited by BART19-3p mimics (Fig. 5F).

Finally, we confirmed that GADD45B was significantly reduced compared with negative control in AGS and GES cells overexpressing EBV-miR-BART19-3p by protein levels (Fig. 5G). While FOSB and CCDC80 protein level was not reduced in AGS and GES cells by overexpressing EBV-miR-BART19-3p Supplementary Fig. 3). In contrast, GADD45B protein level was increased with transfection of EBV-miR-BART19-3p antagomir into AGS EBV cells (Fig. 5G). Since EBV-miR-BART19-3p overexpression can promote proliferation and reduce the ratio of G2/M phase in AGS and GES1 cell lines, we further detected proliferation associated proteins (Ki67, PCNA) and G2/M phase associated proteins (cyclin B1, CDK1). It was confirmed by Western blotting that protein levels of Ki67, PCNA, and cyclin B1, but not CDK1, were further elevated after overexpression of EBV-miR-BART19-3p. Similarly, Knockdown of EBV-miR-BART19-3p attenuated protein expression of Ki67, PCNA, and cyclin B1; however, it had no effect on CDK1 (Fig. 5G).

### Restored GADD45B rescues the phenotypes of EBV-miR-BART19-3p overexpression

**Fig. 6:**
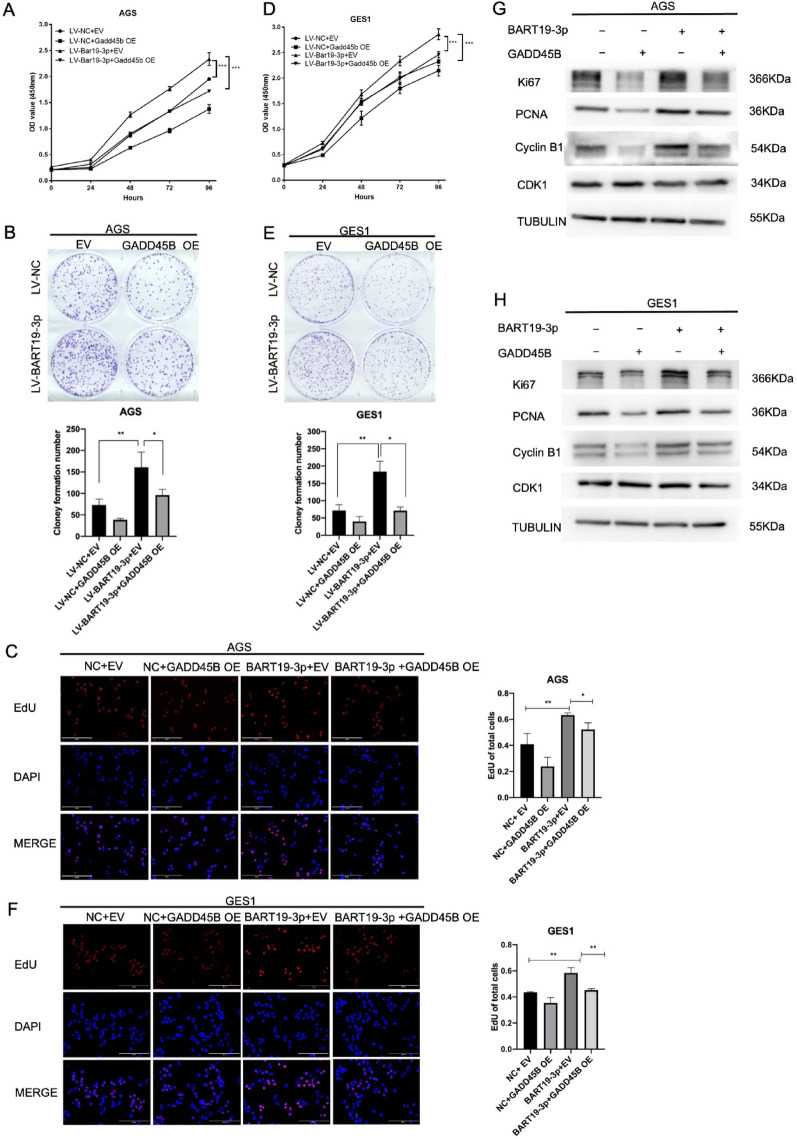
Restored GADD45B rescues the phenotypes of EBV-miR-BART19-3p. Stable EBV-miR-BART19-3p overexpressing AGS cells were transfected with GADD45B plasmids or negative control for 48 h and then subjected to CCK8 assay (**A**), Colony-forming assay (**B**) and EdU incorporation assay (**C**). Stable EBV-miR-BART19-3p overexpressing GES-1 cells were transfected with GADD45B plasmids or negative control for 48 h and then performed by CCK8 assay (**D**), Colony-forming (**E**), and EdU incorporation assay (**F**). (**G**,** H**) Protein levels of GADD45B, Ki67, PCNA, cyclin B1, and CDK1 were detected by Western blot with Tubulin as an internal reference in Stably EBV-miR-BART19-3p overexpressing AGS cells (**G**) and Stable EBV-miR-BART19-3p overexpressing GES-1 cells (**H**) after transfected with GADD45B plasmids or negative control for 48 h. Data are shown as mean ± SD of three experiments, *p < 0.05, **p < 0.01, ***p < 0.001

To confirm that EBV-miR-BART19-3p plays a tumor growth role by inhibiting GADD45B, we transfected the GADD45B expression vector into AGS and GES cells (Supplementary Fig. 4). The result of CCK8 (Fig. 6A, B), clone formation (Fig. 6C, D), and EdU (Fig. 6E, F) showed that GADD45B overexpression could rescue the promote proliferation effect of EBV-miR-BART19-3p in ASG and GES1 cells compared to the GADD45B negative control. Moreover, western blotting results showed that reinstallation of GADD45B attenuated protein levels of proliferation markers and G2/M cell cycle markers upregulated by EBV-miR-BART8-3p compared with GADD45B negative control (Fig. 6G, H).

## Discussion

Epstein-Barr virus is the first virus found to generate miRNAs in 2004[31]. Up to now, 44 mature EBV miRNAs have been identified according to miRBase (https://www.mirbase.org/). Multiple EBV BART miRNAs have been testified to be abnormally highly expressed in EBV-related malignant tumors, promote tumor cell proliferation, invasion and migration, and resist cell apoptosis [32]. EBVaGC is a malignant tumor between latency I and latency II with abundant expression of EBV BART miRNAs [20].

In our study, we detected 5 cases of EBV-positive gastric cancer tissues and found that EBV-miR-BART19-3p was highly expressed in the tumors while absent in paired normal gastric tissues, which confirmed that EBV-miR-BART19-3p originates from the genome of Epstein-Barr virus and the role of Epstein-Barr virus in promoting cancer. EBV-miR-BART19-3p was reported upregulated in EBV-associated diseases, including chronic active EBV infection, EBV-associated hemophagocytic lymphohistiocytosis and nasopharyngeal carcinoma [28, 33]. Xia He.etal detected all 44 mature EBV BART miRNAs in plasma of 159 NPC patients and 145 normal controls by RT-qPCR, only EBV-miR-BART19-3p was significant in all NPC patients, suggesting that plasma EBV-miR-BART19-3p may become a diagnostic biomarker for nasopharyngeal carcinoma [34].

In our study, we overexpressed and knocked down BART19-3p expression in both EBV negative and EBV positive gastric cancer cells, and the results showed that BART1 greatly endorses tumor proliferation both in vitro and in vivo by affecting cell cycle. Upregulation of EBV miR-BART19-3p can promote the proliferation of human immortalized gastric epithelial cells and gastric cancer cells and reduce the number of cells in G2/M phase arrest; however, no significant effect on apoptosis, probably because the apoptosis rate of the gastric tumor cells was too low to make a significant statistical difference. It has been reported that transfection of miR-BART19-3p mimics into EBV-negative leukemia cell line Jurkat and EBV-negative nasopharyngeal carcinoma cell line CNE2 can promote cell proliferation [33].

A few EBV BART miRNAs have been identified in EBVaGC. EBV-miR-BART5-3p, EBV-miR-BART4-5p, and EBV-miR-BART20-5p promote the growth and inhibit the apoptosis of gastric carcinoma cells by directly targeting the tumor suppressor gene TP53 3'-UTR, BID 3'-UTR and the 3'-UTR of BAD, respectively [14, 21, 35]. However, EBV-miR-BART1-3p could induce G0/G1 arrest and inhibit cell growth in gastric carcinoma cells by targeting E2F3 mRNA and regulating miR-17-92 cluster [36], and has even been detected in exosome of EBVaGC [37]. Choi etal reported EBV-miR-BART15-3p could induce apoptosis in AGS-EBV cells by inhibiting the translation of the apoptosis inhibitor BRUCE [38]. EBV-miR-BARTs also play a key role in promoting epithelial mesenchymal transition (EMT) in nasopharyngeal carcinoma, another EBV-associated epithelial origin malignant tumor. EBV-miR-BART1 and EBV-miR-BART7-3p can induce EMT and drive tumor metastasis by directly targets the tumor cell suppressor PTEN and affecting its downstream signal pathways in NPC cells [28–39]. Additionally, EBV-miR-BART13 can directly target NF-κB inhibitor interacting Ras-like 2 (NKIRAS2) to promote growth and metastasis in NPC [27]. miR-BART5-5p overexpression in EBV negative gastric cancer cells caused a reduction of PIAS3 and then STAT3 stimulation followed by PD-L1 upregulation [40].

To probe the mechanisms of EBV-miR-BART19-3p in the proliferation of EBVaGC, we exploited analysis of RNA-deep sequencing and bioinformatics prediction to search for its candidate downstream genes. With the further validation of dual-luciferase reporter assay and Western Blotting in cell lines, GADD45B was finally screened as the major target of EBV-miR-BART19-3p in EBVaGC. EBV-miR-BART19-3p is also reported to target APC in NPC, gastric cancer, and EBV positive diffuse large B-cell lymphom a [33, 34]. GADD45B (growth arrest and DNA damage-inducing protein GADD45 Beta) is a member of the GADD45 gene family. It forms an evolutionarily conserved family of small acidic nucleoproteins with GADD45A and GADD45G, and together inhibits cell proliferation and growth [41–43]. YM Cheng found [44] that sulforaphane may dissociate the cyclin B1/CDK1 complex by up-regulating GADD45B protein, thereby arresting in the G2/M phase and inhibiting the proliferation of cervical cancer. Vairapandi [45] reported that Gadd45b could interact with cyclin B1 and Cdk1, resulting in disruption of the cyclinB1/Cdk1 complex and thus inhibiting the complex kinase activity. In this study, we found overexpression of EBV-miR-BART19-3p inhibited GADD45B and reduced arrest in the G2/M phase of EBVaGC cells. Consistently, our results showed that overexpression of GADD45B can down-regulate the protein expression of cyclin B1, but not CDK1.

There are still some limitations in our study. First, the number of EBVaGC samples collected in this study is relatively small. More gastric cancer specimens can be collected to expand EBV-positive gastric cancer cases. Secondly, the study confirmed that EBV-miR-BART19-3p directly targeted GADD45B and regulated the protein level of cyclinB1 by Western Blot. However, further experiments are needed to clarify the direct molecular mechanisms between GADD45B and cyclin B1/CDK1 complex in AGS EBV cells, which can be explored in future studies. Silencing of oncogenic EBV-miR-BART19-3p by RNA interference might be a potential therapeutic target of EBVaGC in clinical application in the future.

**Fig. 7:**
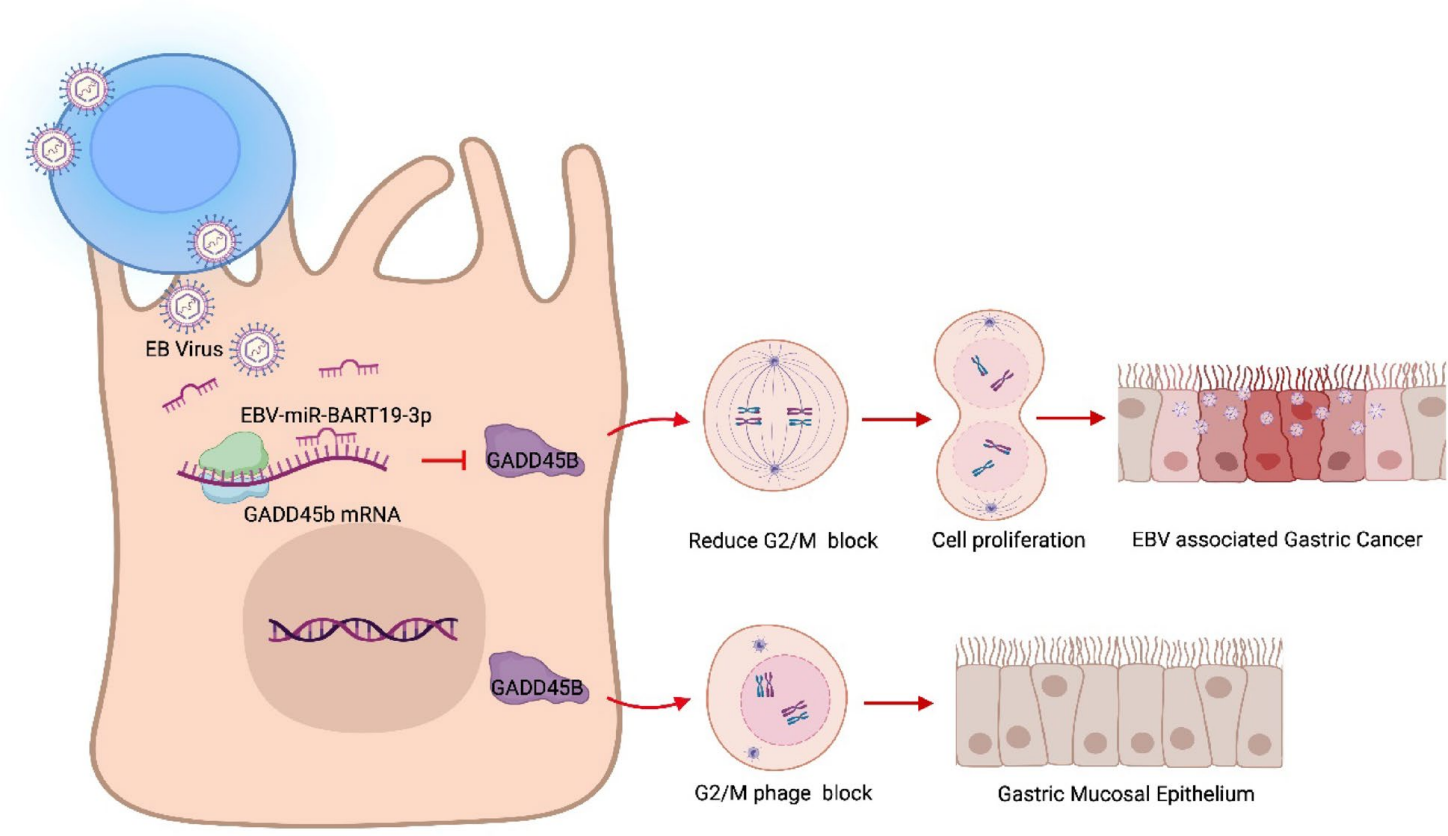
EBV-miR-BART19-3p promotes proliferation of EBV-associated Gastric Cancer by inhibiting GADD45B. The gastric mucosal epithelial cell was infected EB virus by B lymphocytes cell contacts, then BART19 was highly expressed and released into its cytoplasm. EBV-miR-BART19-3p directly targets the growth arrest and DNA damage-inducing protein Beta mRNA (GADD45B) and downregulates its protein expression of host epithelial cell which consequently reduce cell cycle G2/M phase arrest. EBV-miR-BART19-3p can promote the proliferation of mucosal epithelial cell and lead to tumor formation of EBVaGC

In conclusion, we revealed that EBV-miR-BART19-3p promoted the proliferation and reduced the cell G2/M arrest of EBVaGC cells and immortalized gastric epithelial cells by directly suppressing GADD45B (Fig. 7). Upregulation of GADD45B can rescue the proliferation generated by EBV-miR-BART19-3p. Our study reveals a new mechanism for EBV manipulation of cell growth in EBVaGC, indicating that inhibition of EBV-miR-BART19-3p may offer a new therapeutic approach for EBVaGC patients.

## Supplementary Information

Below is the link to the electronic supplementary material.

Supplementary Material 1

## Data Availability

The dataset supporting the conclusions of this article is available in the GEO database (GSE302909) with link https://www.ncbi.nlm.nih.gov/geo/query/acc.cgi?acc=GSE302909.

## Abbreviations

GC: Gastric cancer
TCGA: The Cancer Genome Atlas
EBVaGC: Epstein-Barr virus associated gastric cancer
EBNA1: EBV encoded nuclear antigen 1
EBV: Epstein-Barr virus
BART miRNAs: Bam-HI-A rightward transcripts miRNAs
BARF1 miRNA: Bam-HI A rightward frame 1 microRNA
3′UTR: 3′-Untranslated regions
5′UTR: 5′-Untranslated regions
APC: Adenomatous polyposis coli
FBS: Fetal bovine serum
qRT-PCR: Quantitative real-time PCR
EBER-ISH: Epstein-Barr encoding region in situ hybridization
CCK-8: Cell counting Kit-8
NPC: Nasopharyngeal carcinoma
EBV-HLH: EBV-associated hemophagocytic lymphohistiocytosis
NGS: Next-generation sequencing
GO: Gene ontology
Cyclin B1: Cell cycle B1
Ki67: Proliferation-related Ki-67 antigen
PCNA: Proliferating cell nuclear antigen
CDK1: Cyclin-dependent kinases 1
GADD45B: Growth arrest and DNA damage inducible beta
Cyclin/CDK: Cyclin/cyclin-dependent kinase complex
CKI: Cyclin dependent kinase inhibitor
