# Supplementary material for: Epstein-Barr virus-coded miR-BART19-3p promotes proliferation of EBV-associated gastric cancer by inhibiting GADD45B
Source: J Transl Med. 2025 Aug 25;23:956. doi: 10.1186/s12967-025-06955-9 (PMC12376731; doi:10.1186/s12967-025-06955-9)
Supplement: Supplementary file 1 — Supplementary Material 1 [file 12967_2025_6955_MOESM1_ESM.docx]

**Supplementary to: Epstein-Barr virus-coded miR-BART19-3p promotes proliferation of EBV-associated Gastric Cancer by inhibiting GADD45B**

Supplementary Table 1. The primers of genes

| Primer | Sequence |
| --- | --- |
| EBNA1-F for PCR | CGTCTTACACCATTGAGTCGTCTCC |
| EBNA1-R for PCR | GCGGGCTTTGGCATAACAAGGT |
| EBV-miR-BART19-3p-F | CGCGTTTTGTTTGCTTGGG |
| EBV-miR-BART19-3p-R | AGTGCAGGGTCCGAGGTATT |
| EBV-miR-BART19-3p stem-loop primer | GTCGTATCCAGTGCAGGGTCCGAGGTATTCGCACTGGATACGACAGCATT |
| U6-F | CTCGCTTCGGCAGCACA |
| U6-R | AACGCTTCACGAATTTGCGT |
| GAPDH-F | CCATGGGGAAGGTGAAGGTC |
| GAPDH-R | TGATGACCCTTTTGGCTCCC |
| ATF3-F | CCAGCAGCAGAGAACCATCAAG |
| ATF3-R | ATGAAAGGCGGGCAGGACAC |
| RASD1-F | CACCGCAAGTTCTACTCCATCC |
| RASD1-R | GGTTGTCCAGACTGAACACCAG |
| FOSB -F | GTGCGAGTGTGAGAGTGTGA |
| FOSB -R | GCAACAGTGCAGAACCAAGG |
| GADD45B-F | GCCAGGATCGCCTCACAGTGG |
| GADD45B-R | GGATTTGCAGGGCGATGTCATC |
| CCDC80-F | GCTGGTGATCTCTGCTCCTAAC |
| CCDC80-R | CTTCCTCTCCAACGCCTAAAAGC |

Supplementary Table 2. The sequence of mimic and antagomir

| Products |  | Sequence |
| --- | --- | --- |
| EBV-miR-BART19-3p mimic | sense | UUUUGUUUGCUUGGGAAUGCU |
|  | antisense | AGCAUUCCCAAGCAAACAAAA |
| mimic negative control | sense | UUUGUACUACACAAAAGUACUG |
|  | antisense | CAGUACUUUUGUGUAGUACAAA |
| EBV-miR-BART19-3p antagomir | sense | AGCAUUCCCAAGCAAACAAAA |
| antagomir negative control | sense | CACAAAUUCGGAUCUACAGGGUA |

Supplementary Table 3. Clinicopathological profiles of gastric cancer patients

| Clinical characteristics | EBVaGC  （N=5） | Non-EBVaGC  （N=90） | *P* value |
| --- | --- | --- | --- |
| Age（years）  ≤60  >60 | 2  3 | 18  72 | 0.2826 |
| Gender  Male  Female | 4  1 | 51  39 | 0.5732 |
| Tumor size （cm）  ≤3  >3 | 1  4 | 38  52 | 0.6455 |
| Histologic differentiation  Poor undifferentiated  moderate differentiated  Well differentiated | 2  3  0 | 32  39  19 | 0.5017 |
| TNM stage  I+II  III+IV | 3  2 | 53  37 | >0.999 |

Supplementary Table 4: List of significantly difference genes

| Gene name | Gene id | Gene_biotype | BaseMean | log_2_FC | *P* adj |
| --- | --- | --- | --- | --- | --- |
| SLAMF7 | ENSG00000026751 | protein_coding | 29.9129511 | 2.30939896 | 0.00762536 |
| HDAC9 | ENSG00000048052 | protein_coding | 58.0446274 | 2.6824001 | 0.00108371 |
| CCDC80 | ENSG00000091986 | protein_coding | 259.900174 | 2.67414337 | 0.00093304 |
| GADD45B | ENSG00000099860 | protein_coding | 355.767325 | 2.04308879 | 3.2508E-08 |
| GRIK5 | ENSG00000105737 | protein_coding | 32.0868095 | 2.21847966 | 0.01111878 |
| RASD1 | ENSG00000108551 | protein_coding | 17.978823 | 4.3938723 | 0.00028115 |
| IL1RL1 | ENSG00000115602 | protein_coding | 6.63051784 | 5.20710746 | 0.03516038 |
| NPPB | ENSG00000120937 | protein_coding | 19.5082587 | 2.68840653 | 0.00420494 |
| TEX14 | ENSG00000121101 | protein_coding | 73.0909934 | 3.31569444 | 1.7121E-11 |
| FOSB | ENSG00000125740 | protein_coding | 148.419682 | 3.33612119 | 0.00455128 |
| MYPN | ENSG00000138347 | protein_coding | 13.276259 | 3.40847934 | 0.02663315 |
| SYT16 | ENSG00000139973 | protein_coding | 6.6637093 | 6.21142576 | 0.0079064 |
| RPL3L | ENSG00000140986 | protein_coding | 13.7469481 | 3.07604516 | 0.00733654 |
| TRIM43B | ENSG00000144010 | protein_coding | 4.4403138 | 5.62534297 | 0.03751614 |
| CLTRN | ENSG00000147003 | protein_coding | 11.3877577 | 2.76509777 | 0.02201781 |
| GOLGA6A | ENSG00000159289 | protein_coding | 14.0622827 | 4.03015402 | 0.00234096 |
| DDR2 | ENSG00000162733 | protein_coding | 77.9818422 | 2.96341393 | 7.6119E-06 |
| ATF3 | ENSG00000162772 | protein_coding | 1476.47152 | 2.53689017 | 5.7955E-18 |
| IL24 | ENSG00000162892 | protein_coding | 10.6150423 | 3.2922635 | 0.01302128 |
| BEST1 | ENSG00000167995 | protein_coding | 23.9600442 | 2.36232353 | 0.00166509 |
| ACTBL2 | ENSG00000169067 | protein_coding | 32.7958529 | 3.26968316 | 7.734E-05 |
| CXCL8 | ENSG00000169429 | protein_coding | 21.5978159 | 2.25122122 | 0.00347017 |
| ITGAM | ENSG00000169896 | protein_coding | 14.4869426 | 2.68857059 | 0.02863965 |
| FOS | ENSG00000170345 | protein_coding | 119.993795 | 2.32260122 | 5.7882E-06 |
| GPC5 | ENSG00000179399 | protein_coding | 8.05237051 | 3.94964775 | 0.02871066 |
| ARL14 | ENSG00000179674 | protein_coding | 19.9433194 | 2.11554789 | 0.03390337 |
| BPIFC | ENSG00000184459 | protein_coding | 8.840437 | 4.08292528 | 0.03360481 |
| GAST | ENSG00000184502 | protein_coding | 20.9302699 | 3.40805981 | 0.00286129 |
| CLDN6 | ENSG00000184697 | protein_coding | 10.0274625 | 4.89434644 | 0.01329089 |
| Gene name | Gene id | Gene_biotype | BaseMean | log_2_FC | padj |
| IGFL3 | ENSG00000188624 | protein_coding | 7.4952189 | 4.45673497 | 0.0486983 |
| SERPINB2 | ENSG00000197632 | protein_coding | 41.579687 | 2.66074523 | 0.00143826 |
| RNU5A-1 | ENSG00000199568 | snRNA | 31.9714997 | 6.61045798 | 4.6612E-06 |
| RNU5D-1 | ENSG00000200169 | snRNA | 5.91179183 | 6.03965793 | 0.0050657 |
| RNVU1-6 | ENSG00000201558 | snRNA | 10.9304473 | 3.1067542 | 0.01623837 |
| RNU4-2 | ENSG00000202538 | snRNA | 10.7939051 | 3.94703756 | 0.00595262 |
| SPANXN5 | ENSG00000204363 | protein_coding | 4.89254324 | 5.76686615 | 0.01282595 |
| FRG2 | ENSG00000205097 | protein_coding | 10.1107119 | 5.82684923 | 0.014413 |
| RNVU1-7 | ENSG00000206585 | snRNA | 93.3049342 | 9.05709669 | 7.1465E-10 |
| RNU1-27P | ENSG00000206596 | snRNA | 5.58270663 | 4.95240321 | 0.04414672 |
| RNU1-1 | ENSG00000206652 | snRNA | 11.6702452 | 3.44498402 | 0.01707334 |
| KRTAP3-1 | ENSG00000212901 | protein_coding | 83.7700488 | 2.55688529 | 1.3901E-12 |
| BX546450.1 | ENSG00000214915 | lncRNA | 16.5165453 | 6.54624456 | 0.00057247 |
| USP17L2 | ENSG00000223443 | protein_coding | 46.4117362 | 6.55607912 | 1.0036E-06 |
| RFPL4A | ENSG00000223638 | protein_coding | 120.326371 | 7.51752373 | 3.6497E-05 |
| LINC02158 | ENSG00000225611 | lncRNA | 16.349476 | 4.5837034 | 0.00183644 |
| USP17L7 | ENSG00000226430 | protein_coding | 44.1480045 | 7.08302498 | 0.00726459 |
| RFPL4AL1 | ENSG00000229292 | protein_coding | 12.3088089 | 6.11706789 | 0.00455128 |
| LINC00452 | ENSG00000229373 | lncRNA | 5.77374694 | 4.99920531 | 0.04378633 |
| AC004835.1 | ENSG00000230392 | lncRNA | 10.4573512 | 4.34409898 | 0.03922065 |
| AL353743.4 | ENSG00000234424 | unprocessed_pseudogene | 9.12639413 | 3.69374564 | 0.02492056 |
| RNF148 | ENSG00000235631 | protein_coding | 6.9185095 | 4.35648721 | 0.04986822 |
| LINC01447 | ENSG00000236078 | lncRNA | 7.99865558 | 4.53402686 | 0.04389482 |
| USP17L8 | ENSG00000237038 | protein_coding | 5.11333568 | 5.82910454 | 0.01791598 |
| AC008897.2 | ENSG00000247372 | lncRNA | 13.0262171 | 7.17913076 | 0.00038601 |
| MCPH1-AS1 | ENSG00000249898 | lncRNA | 25.357964 | 2.01475332 | 0.02603712 |
| AC087203.1 | ENSG00000254423 | processed_pseudogene | 12.1005487 | 4.16667863 | 0.03296872 |
| LINC02757 | ENSG00000255363 | lncRNA | 9.17638334 | 4.15172948 | 0.02197972 |
| AC105129.2 | ENSG00000259346 | processed_pseudogene | 10.5241579 | 4.98366547 | 0.01254784 |
| LINC02533 | ENSG00000260239 | lncRNA | 6.27745009 | 6.1256417 | 0.00524971 |
| SNORD3B-2 | ENSG00000262074 | snoRNA | 10.7657373 | 3.61400264 | 0.00948635 |
| AC007952.4 | ENSG00000262202 | lncRNA | 70.1976165 | 3.65111388 | 1.2735E-10 |
| SNORD3A | ENSG00000263934 | snoRNA | 45.6308033 | 3.49070474 | 6.9307E-11 |
| RNU4ATAC | ENSG00000264229 | snRNA | 14.4685044 | 4.84656282 | 0.00044353 |
| SNORD3B-1 | ENSG00000265185 | snoRNA | 42.000288 | 2.8801689 | 1.8402E-08 |
| AC012447.1 | ENSG00000265451 | lncRNA | 37.0477426 | 5.22018182 | 1.5347E-09 |
| AC243960.3 | ENSG00000268355 | lncRNA | 25.5033025 | 2.95066249 | 0.00108371 |
| AC012313.8 | ENSG00000269473 | lncRNA | 25.3035792 | 2.119286 | 0.02965593 |
| Z93241.1 | ENSG00000270022 | lncRNA | 17.6304841 | 5.13748698 | 8.519E-05 |
| RNVU1-31 | ENSG00000270722 | snRNA | 66.3957866 | 9.53011511 | 7.3225E-11 |
| H2AC19 | ENSG00000272196 | protein_coding | 28.9601987 | 2.02385429 | 0.02383705 |
| AP000692.2 | ENSG00000273199 | lncRNA | 13.1725466 | 3.00521538 | 0.00312185 |
| RNVU1-29 | ENSG00000273768 | snRNA | 13.3952174 | 7.22166918 | 2.5767E-05 |
| TAF11L11 | ENSG00000283740 | protein_coding | 9.8586743 | 6.7770433 | 0.00057227 |

Supplementary Fig 1. Validation of AGS, GES1 cell lines stable transfection with EBV-miR-BART19-3. A, Fluorescent photograph of AGS and GES1 cells after infection with LV-NC or LV-BART19-3p lentiviral vector, magnification 100×. B, the expression of EBV-miR-BART19-3p expression in AGS and GES1 stably transfected cell lines was detected by RT-qPCR, with EBV-positive lymphoma Raji cells as a positive reference (n=3. Means±SD, ***p<0.001)

Supplementary Fig 2. Effect on apoptosis of overexpression of EBV-miR-BART19-3p in AGS and GES1 cells. Apoptosis rates of LV-NC groups and LV-BART19-3p in AGS (A) and GES1 (B) cell lines were detected by flow cytometry after Annexin V-PE/7-AAD double staining.


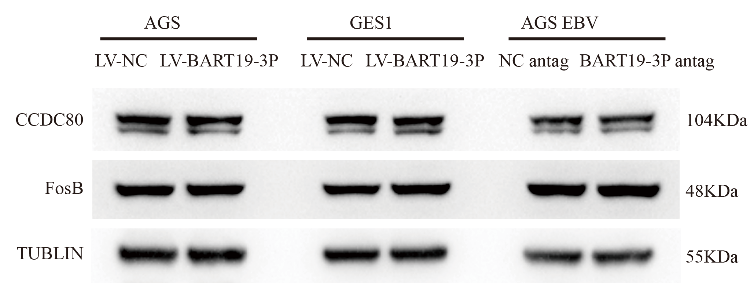


Supplementary Fig 3. Protein levels of CCDC80 and FosB were detected by Western Blot with Tubulin as an internal reference.

Supplementary Fig 4. Verification of GADD45B in GES1 and AGS cells by Western Blot. A: GADD45B protein levels were detected by Western Blot in GES1 (A) and AGS (B) cells transfected with pcDNA3.1-Empty Vector, pcDNA3.1-GADD45B OE and blank control group, respectively. (n=3, Means±SD, ***p<0.001)
